# NF-κB/TWIST1 Mediates Migration and Phagocytosis of Macrophages in the Mice Model of Implant-Associated *Staphylococcus aureus* Osteomyelitis

**DOI:** 10.3389/fmicb.2020.01301

**Published:** 2020-06-12

**Authors:** Yutian Wang, Yihuang Lin, Caiyu Cheng, Pengyu Chen, Ping Zhang, Hangtian Wu, Kaiqun Li, Ye Deng, Jikun Qian, Xianrong Zhang, Bin Yu

**Affiliations:** ^1^Department of Orthopaedics, Nanfang Hospital, Southern Medical University, Guangzhou, China; ^2^Guangdong Provincial Key Laboratory of Bone and Cartilage Regenerative Medicine, Nanfang Hospital, Southern Medical University, Guangzhou, China

**Keywords:** *Staphylococcus aureus*, osteomyelitis, macrophage, bioinformatics, bone remodeling

## Abstract

*Staphylococcus aureus* (*S. aureus*) infection-induced osteomyelitis is a great challenge in clinic treatment. Identification of the essential genes and biological processes that are specifically changed in mononuclear cells at an early stage of *S. aureus* osteomyelitis is of great clinical significance. Based on transcriptional dataset GSE16129 available publicly, a bioinformatic analysis was performed to identify the differentially expressed genes of osteomyelitis caused by *S. aureus* infection. ERBB2, TWIST1, and NANOG were screened out as the most valuable osteomyelitis-related genes (OMRGs). A mice model of implant-associated *S. aureus* osteomyelitis was used to verify the above genes. We found significantly up-regulated expression of TWIST1 in macrophages and accumulation of macrophages around the infected implant. Meanwhile, *S. aureus* infection increased the expression of TWIST1, MMP9, and MMP13, and stimulated the migration and phagocytosis function of Raw 264.7 cells. Additionally, knock-down of the expression of TWIST1 by siRNA could significantly down-regulate MMP9 and MMP13 and suppress the migration and phagocytosis ability of macrophages in response to *S. aureus* infection. Furthermore, we found that NF-κB signaling was activated in Raw 264.7 cells by *S. aureus* and that inhibition of NF-κB signaling by Bay11–7082 blocked the expression of TWIST1, MMP9, and MMP13 as well as cell migration and phagocytosis evoked by *S. aureus*. Our findings demonstrate that NF-κB/TWIST1 is necessary for migration and phagocytosis of macrophages in response to *S. aureus* infection. Our study highlights the essential role of NF-κB/TWIST1 in early innate immune response to *S. aureus* infection in bone.

## Introduction

Osteomyelitis is an inflammatory process in bone resulting from infection by such micro-organisms as bacteria, fungi, or mycobacteria (Xiong and Pamer, 2015; Momodu and Savaliya, 2019). Its incidence in the pediatric population is approximately 5–8/10,000 each year (Riise et al., 2008; Peltola and Paakkonen, 2014). Its major etiology can be hematogenous, tracking from adjacent foci of infection, and direct inoculation from trauma or surgery (Kavanagh et al., 2018). An acute process is considered when the duration of symptoms is less than 2 weeks (Yeo and Ramachandran, 2014). Acute hematogenous osteomyelitis usually affects the skeleton in children as the metaphysis of a growing long bone contains abundant blood vessels with leaky endothelium and sluggish blood flow, which is commonly the primary site of infection (Stephen et al., 2012; Whyte and Bielski, 2016). Delay in diagnosis and inappropriate treatment may lead to septicaemia and intense inflammatory reaction, which destroys bone structures with longitudinal growth arrest or bone defects, and may even bring about deteriorated outcomes of multiorgan failure, and death (Fink et al., 1977).

As the most common causative pathogen of osteomyelitis (Peltola and Paakkonen, 2014), *Staphylococcus aureus* (*S. aureus*) infection can trigger strong inflammatory response through its virulence factors and structural components (Bogoslowski et al., 2018; Putnam et al., 2019), leading to a substantial bone loss during osteomyelitis (Mbalaviele et al., 2017). Peripheral blood mononuclear cells (PBMCs), the precursors of dendritic cells, osteoclasts and macrophages, are key actors recruited which exert an immediate and potent immune response against *S. aureus* infection, playing an important role in inflammatory bone loss (Xiong and Pamer, 2015; de Vries et al., 2019). Subjects with suppressed monocytes/macrophages may have an increased susceptibility to *S. aureus* infection (Knobloch et al., 2019), whereas early accumulation of inflammatory monocytes/macrophages may serve as a reservoir for intracellular *S. aureus* survival, thereby promoting bacterial resistance to antibiotic treatment (Fischer et al., 2019). In a previous study, we demonstrated that the G-CSF-mediated bone loss might be due to aggregation of F4/80^+^ macrophages (Hou et al., 2019). However, the events of migration and immune response by macrophages at an acute stage of *S. aureus* infection in bone have been poorly understood. Clarification of macrophage migration in response to *S. aureus* infection in bone is essential for identification of targets in treatment of osteomyelitis induced by *S. aureus* infection.

High-throughput transcriptional analysis is a useful technique to investigate the multidimensional networks of molecules and cells in response to stimulus (Kulasingam and Diamandis, 2008). In the present study, we downloaded the transcriptome profiles of GSE16129 (Ardura et al., 2009) and analyzed the differentially expressed genes (DEGs) in mononuclear/macrophage cells between *S. aureus* osteomyelitis patients (median age 7.5 years) and healthy controls (median age 6 years) using bioinformatics methods. Finally, TWIST1, NANOG, and ERBB2 were screened out as the DEGs most likely related to immunity of marrow and bone metabolism. Our study revealed that *S. aureus* infection might stimulate NF-κB/TWIST1 signaling, thereby promoting migration and phagocytosis of macrophages.

## Materials and Methods

### Microarray Data Preprocessing

The genes expression profile GSE16129 from the study by Ardura et al. (2009) was downloaded from the publicly available gene expression omnibus database^1^ based on three platforms (GPL96, GPL97, and GPL6106). A bioinformatic analysis was performed. As there was only one osteomyelitis sample with *S. aureus* infection in the GPL6106 platform, we did not include it in the present study. In these platforms, the total RNA extracted from PBMCs of healthy controls and patients was utilized for gene expression microarrays. R statistical software (version 3.5.2, R Project for Statistical Computing^2^) was used to perform the analysis process.

### Analysis and Screen of DEGs

**FIGURE 1:**
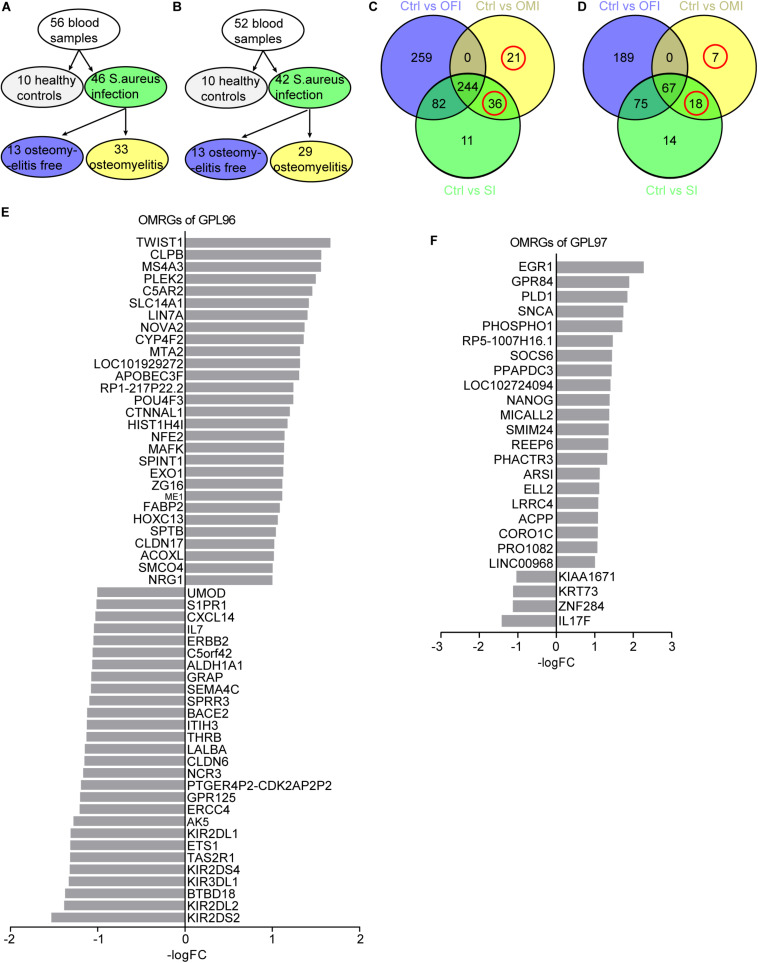
Sample grouping and DEGs from OFI, OMI, and SI groups. **(A,B)** Grouping plots of transcriptional file from platform GPL96 and GPL97. **(C,D)** Venn diagram of DEGs and OMRGs of GPL96 and GPL97. Red circle represents the OMRGs. **(E,F)** All the up-regulated and downregulated OMRGs of GPL 96 and GPL97. The horizontal axis shows the -log (fold change), and the vertical axis represents gene names.

To further analyze the genes related to *S. aureus* osteomyelitis, three groups comparing healthy control (Ctrl) vs osteomyelitis-free infection (OFI), Ctrl vs osteomyelitis infection (OMI) and Ctrl vs *S. aureus* infection (SI; Figures 1A,B) were analyzed by limma package^3^ (Diboun et al., 2006) and R statistical software (version 3.5.2). In order to reduce the false positive rate, the *P*-value was adjusted using Benjamini and Hochberg false discovery rate method. Only genes with |log2 FC (fold change)| > 1 and adjusted *P*-value < 0.05 were identified as the DEGs. To screen the overlapping and unique genes in three comparison groups, Venn diagram^4^ was used to analyze different DEGs (Figures 1C,D). The DEGs observed from Ctrl vs. OMI but not from Ctrl vs. OFI were considered as osteomyelitis-related genes (OMRGs).

### Gene Ontology (GO) Functional Annotation, Kyoto Encyclopedia of Genes and Genomes (KEGG) Enrichment Analysis, Protein-Protein Interaction (PPI) Network Analysis and Module Identification

The database for annotation, visualization and integrated discovery (DAVID)^5^ was used to perform GO functional annotation (Ashburner et al., 2000), and KEGG pathway enrichment analysis (Kanehisa et al., 2008) of the OMRGs. The significantly enriched GO terms and KEGG pathways with the thresholds of *P*-value <0.05 were selected for analyzing *S. aureus* osteomyelitis-associated biological process and pathways. The online tool STRING database^6^ was used to establish the PPI network and identify the key proteins and important protein modules (Szklarczyk and Jensen, 2015). The Cytoscape software (version 3.6.1) was used to analyze degree of distribution, closeness centrality and betweenness centrality. The nodes that owned a relatively large number of edges (interactions) were identified as core proteins that play a major role in the PPI network. The top 15 core proteins with a high degree of relatedness were selected. Then, the module analysis was conducted with the MCODE plugin of Cytoscape software with thresholds of degree cutoff = 2, node score cutoff = 0.2, k-core ≥ 2, and max. depth = 100 (Bader and Hogue, 2003).

### Bacterial Strain and Preparation

*Staphylococcus aureus* was isolated from the osteomyelitis patients in Nanfang Hospital and identified using PHOENIX 100 (Becton Dickinson Microbiology System, United States). For the infection experiments, *S. aureus* was inoculated in 10 ml fresh tryptic soy broth (TSB) and incubated overnight at 37°C with shaking at 200 rpm/min. Bacteria were collected by centrifugation, washed three times with phosphate-buffered saline (PBS), and re-suspended in PBS. The concentration of *S. aureus* was adjusted to an OD of 0.5 at 600 nm, approximately equal to 1 × 10^8^ CFU/ml, and then diluted to different concentrations for infection in mice osteomyelitis model or in Raw264.7 macrophage cell line.

### Animals

Eight-week old male C57BL/6 mice were purchased from Laboratory Animal Center of Southern Medical University (Guangzhou, China). Mice were housed under specific pathogen-free conditions at 24–26°C with a 12-h light/dark cycle and access to food and water *ad libitum*. All interventions and animal care procedures were performed according to the Guidelines and Policies for Animal Surgery provided by Nanfang Hospital. The osteomyelitis model used in this study was modified from that reported by Bernthal et al. (2010). Briefly, after anesthesia by intraperitoneal injection of tribromoethanol and sterilization, a 5 mm incision was made along the dorsal side of right femur to expose the bone surface. A hole on the cortical bone of mid-diaphysis of the femur was drilled with a 25-gage syringe needle, without penetrating the cortical bone on the other side. Next, a 2 mm-long sterile stainless steel needle (0.3 mm in diameter) was inserted into the bone marrow cavity through the hole. Mice were divided randomly into *S. aureus* osteomyelitis group (*n* = 19) and control group (*n* = 19). In the *S. aureus* osteomyelitis group, 1 × 10^6^ CFU/ml *S. aureus* in 2 μl of PBS was injected into the canal of the bone marrow cavity using a micro syringe (Sangon, China, Wuhan) while the control mice were injected with the same volume of sterile PBS. The hole was sealed with bone wax, and the incisions were closed with 5–0 sutures. Analgesics was given for perioperative analgesia to minimize pain. On day 3 after surgery and infection, animals were killed by cervical vertebra dislocation, and the right femurs were collected for further analysis.

### Histochemistry, Immunohistochemistry, and Immunofluorescence

Femurs were fixed with 4% paraformaldehyde overnight, decalcified in 10% EDTA (pH 7.4) for 4 weeks, and finally embedded in paraffin. Longitudinally oriented 4 μm-thick sagittal sections were cut and processed for staining. From every consecutive ten sections, two were chosen for hematoxylin and eosin (H&E) staining.

Immunohistochemistry staining was conducted according to the standard protocol. After being deparaffinized and rehydrated, sections were incubated with primary antibodies to TWIST1 (Cat. 25465-1-AP, Proteintech, Wuhan, China), and F4/80 (Cat.71299, Cell Signal Technology, MA, United States) overnight at 4°C. After washing in PBS, sections were incubated with a biotinylated secondary antibody and then with an avidin-biotinylated horseradish peroxidase complex (Vectastain ABC Kit, Vector Laboratories, United States) according to the manufacturer’s protocol. Peroxidase activity was revealed by DAB (PW017, Sangon Biotech, Shanghai, China).

For immunofluorescence staining, frozen sections were incubated with primary antibody to TWIST1 (Cat.25465-1-AP, Proteintech, Wuhan, China), and F4/80 (Cat.71299, Cell Signal Technology, MA, United States) overnight at 4°C, followed by incubation with Alexa Fluor 594-conjugated goat anti-rabbit IgG (H + L; Cat.SA00006-4; Proteintech, Rosemont, IL, United States), and Alexa Fluor 488-conjugated goat anti-mouse IgG (H + L; Cat. SA00006-1; Proteintech) for 1 h at room temperature. Nuclei were counterstained with DAPI (E607303–0002; BBI Life Science, Shanghai, China). The sections were observed through a BX63 microscope (Olympus, Tokyo, Japan). To quantify the positive-stained cells adjacent to implant, we randomly chose 2 non-overlapping area in three sections of each mice sample.

### Total RNA Extraction and Real-Time Quantitative PCR (qPCR)

To obtain tissue RNA, bone was homogenized in TRIzol reagent (TaKaRa, Dalian China), followed by total RNA isolation. RNA was then reverse transcribed to cDNA with PrimeScript^TM^ RT reagent Kit (TaKaRa, Dalian, China). qPCR was conducted using the iQ5 (Bio-Rad, Hercules, CA, United States) with SYBRP remix Ex Taq^TM^ (TaKaRa, Dalian, China). The relative expression of genes was normalized to GAPDH and processed by the 2^–ΔΔCt^ method. All primers are listed in Supplementary Table S1.

### Western Blotting

Bone medullary cavity contents were collected and then lysed by RIPA buffer with proteinase inhibitors (KGP250; KeyGen BioTech, Jiangning, China). Equal amounts of protein (30 μg) were resolved by SDS-PAGE on 10% polyacrylamide gels and then transferred to PVDF membrane. After being blocked with 5% BSA solution at room temperature for 1 h and washed with 1 × tris-buffered saline tween (TBST) three times, membranes were then incubated with the primary antibodies to TWIST1 (Cat. 25465-1-AP, Proteintech, Wuhan, China), P65 (Cat. AF5006, Affinity Cincinnati, OH, United States), phospho-P65 (Cat. AF2006, Affinity Cincinnati, OH, United States), MMP9 (Cat. 13667, Cell Signal Technology, MA, United States), and MMP13 (Cat. ab39012, Abcam, Cambridge, MA, United States). The membranes were further incubated with a HRP-conjugated anti-rabbit (ab6721, Abcam, Cambridge, MA, United States), anti-mouse (ab6728, Abcam, Cambridge, MA, United States) secondary antibody for 1 h at room temperature. All blots were developed using ECL (MilliporeSigma, Burlington, MA, United States). Images were captured with Tanon chemiluminescence apparatus (Tanon-5200Multi, Tanon, China, Shanghai).

### Cell Culture and Treatments

Mouse Raw264.7 cell line was purchased from Cell Bank, Shanghai Institute of Biochemistry and Cell Biology at the Chinese Academy of Sciences (Shanghai, China). Cells were seeded at a density of 2 × 10^5^ cells/well in 6-well plate and cultured in high-glucose medium DMEM (HyClone, United States) containing 10% fetal bovine serum (FBS; Gibco, United States). To evaluate the expression of genes in macrophage in response to *S. aureus* infection, cells were serum starved overnight, then infected by *S. aureus* with the multiplicity of infection (MOI) range from 0.01 to 10. After 1 h infection, extracellular *S. aureus* were killed with 20 μg/ml gentamicin (Cat. B20192, Sigma Aldrich, St. Louis, Missouri, United States) for 30 min. After washing with PBS three times, cells were allowed to grow in fresh 10% FBS medium for additional 24 h, and then RNA was harvested for mRNA expression analysis.

To knockdown TWIST1 expression, Raw 264.7 cells (2 × 10^5^ cells/well) were seeded in 6-well plate and cultured for 24 h, and then transient RNA interference was performed following the manufacturer’s instructions. Cells were transfected with small interference RNAs for TWIST1 (siRNAs; siBDM1999A, RIBOBIO, GUANGZHOU, China) using Lipofectamine 3000 (Invitrogen, Carlsbad, California, United States). The sequences of designed siRNAs targeting TWIST1 were as follows: si-TWIST1-1 (CAAGATTCAGACCCTCAAA), si-TWIST1-2 (GATGGCAAGCTGCAGCTAT), and si-Twist1-3 (GACTCCAAGATGGCAAGCT). 24 h after transfection, cells were infected with *S. aureus* at MOI of 0.01 for 1 h. Next, after killing the extracellular *S. aureus* with 20 μg/ml gentamicin for 30 min, cells were allowed to grow in fresh 10% FBS medium for additional 24 h, or 48 h. Then RNA and protein were harvested for further analysis.

To evaluate the role of NF-κB signaling in microphage during *S. aureus* infection, cells were pretreated with 10 μM BAY11-7082 (BAY; Cat. S7352; Selleck, Houston, United States) for 2 h, and then infected with *S. aureus* at a MOI of 0.01 for 1 h. Next, *S. aureus* was killed by adding 20 μg/ml gentamicin for 30 min. Next, cells were washed with PBS three times and cultured in fresh 10% FBS medium for additional 48 h, and then proteins were harvested for western blotting.

### Trypan Blue Staining

Trypan blue staining was used to determine the optimal MOI and observe the viability of Raw 264.7 cells with *S. aureus* infection in different MOI. 5 × 10^5^ cells were plated in 6-well plates. 24 h later, cells were infected with *S. aureus* at MOI of 0.01 to 10 for 1 h. Next, after the extracellular *S. aureus* eliminated by treatment with 20 μg/ml gentamicin for 30 min, cells were cultured with fresh 10% FBS medium. After culture for 24 h, cells were washed with PBS three times, and stained with 0.04% trypan blue (C0040, Solarbio, Beijing, China) for 3 min. The plates were observed through a BX63 inverted microscope (Olympus, Tokyo, Japan) and cells were counted using Image Lab Software (Bio-Rad, CA, United States).

### Macrophage Migration Assay

Macrophage migration was evaluated using wound-healing assay and transwell-based migration assay. For wound-healing assay, Raw264.7 were seeded at 1 × 10^6^ cells/well in 6-well plate and cultured for 12 h, cells were then infected with *S. aureus* at 0.01 MOI. After 1 h infection, cells were treated with 20 μg/ml gentamicin for 30 min to kill the extracellular *S. aureus*. After being washed with PBS for three times, cells were allowed to grow in fresh 5% FBS medium, and a 200-μl micro pipette tip was used to scraped the macrophage monolayer. Wound-healing gap closure was analyzed and captured by Image Lab Software (Bio-Rad, CA, United States) at four time points (0, 24, 48, and 72 h). For transwell-based migration assays, 1 × 10^5^ cells/well infected with *S. aureus* at 0.01 MOI for 1 h were seeded in the 8 μm upper chamber with serum-free medium, the lower chamber were supplied with 500 μl 10% FBS medium. After 24 h of culture, membranes were fixed in 4% paraformaldehyde and stained with the 1% crystal violet solution. The migration cells were counted using Image Lab Software.

### Macrophage Phagocytosis

2 × 10^5^ Raw 264.7 cells were infected with *S. aureus* at a MOI of 0.01 for 1 h, following by treatment with 20 μg/ml gentamicin for 30 min to kill extracellular bacteria. Cells were washed with PBS for three times, followed by lysis with 0.2% Triton. The cell lysis mixture was cultured on TSB agar plates overnight at 37°C. Bacteria colonies were counted and set as N0. To evaluate the phagocytosis of macrophage, after extracelluar bacteria eliminated, cells were allowed to grow in fresh 10% FBS medium for an additional 1 h. Then cells were lysed and cell lysis mixture was grown on TSB agar plates, and bacteria colonies were counted and set as N1. The rate of phagocytosis was calculated as N0/(2 × 10^5^) (%), and the rate of bacterial killing was calculated as (N0-N1)/N0 (%).

### Statistical Analysis

The results were expressed as mean ± S.E.M. The significance of variability was analyzed by two-tailed Student’s *t* test, one-way analysis of variance (ANOVA) followed by Dunnett’s test, or Mann–Whitney *U*-test. Quantification was performed from at least three independent experimental groups. *P* < 0.05 was considered to be significant in all tests. All data were analyzed using SPSS 20 (IBM, NY, United States).

## Results

### Analysis of DEGs From Microarray Data

GPL96 platform consists of 56 samples of PBMCs, with 10 from healthy people, 33 from osteomyelitis patients and 13 from osteomyelitis-free patients after *S. aureus* infection for 4–6 days (Figure 1A). GPL97 platform consists of 52 PBMCs samples with 10 from healthy people, 29 from osteomyelitis patients and 13 from osteomyelitis-free patients after *S. aureus* infection for 4–6 days. (Figure 1B). The GPL96 and GPL97 raw data were normalized (Supplementary Figures S1A,B) and converted into expression values by the robust multi-array average (RMA) algorithm (Irizarry et al., 2003).

As shown in Figure 1C and Supplementary Figure S1C, 57 OMRGs were considered to be differentially expressed among three comparison groups in GPL96 platform, and 25 OMRGs in GPL97 platform (Figure 1D and Supplementary Figure S1D). Specifically, there were 29 up-regulated, and 28 down-regulated OMRGs in GPL96 platform (Figure 1E) and 21 up-regulated and 4 down-regulated OMRGs in GPL97 platform (Figure 1F). Together, we got 82 unique OMRGs in GPL96 and GPL97 platforms, which were selected for further GO and KEGG pathways analysis.

### GO Functional Annotation, KEGG Pathway and PPI Network Analysis of the DEGs

**FIGURE 2:**
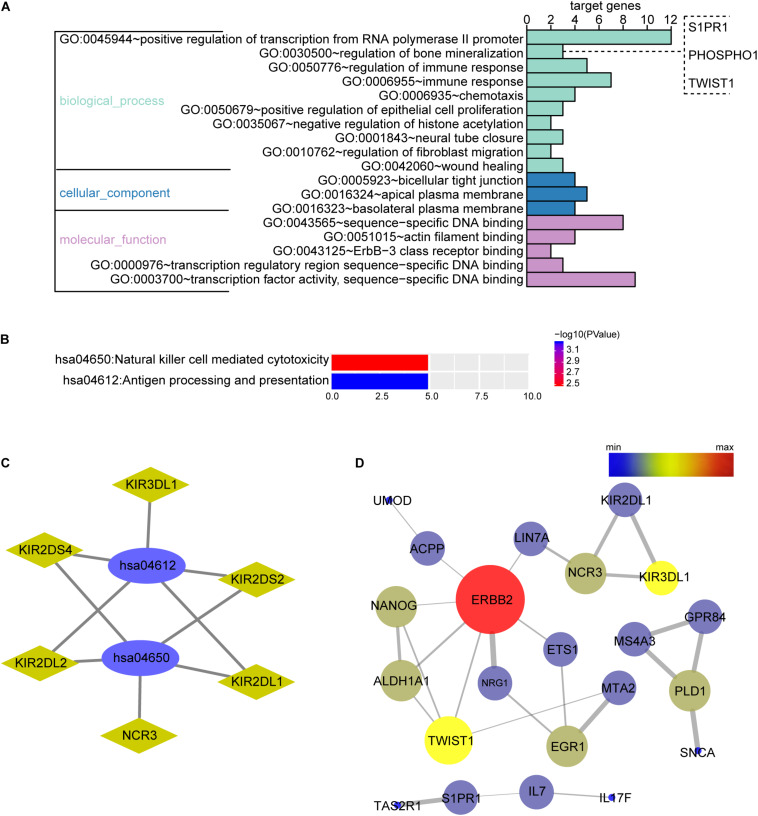
Biological function enrichment analysis of the OMRGs. **(A)** Bar chart of gene ontology (GO) analysis. Genes associated with biological process, cellular component, and molecular function were analyzed for OMRGs. The horizontal axis shows the number of the target genes, and the vertical axis represents GO ID and annotation. **(B)** Bar chart of the KEGG analysis for OMRGs. The horizontal axis shows the number of the target genes, the right vertical axis -log10 (*P* value), and the left vertical axis KEGG pathway ID and annotation. **(C)** Plots show the relationship between the OMRGs and the KEGG pathways. The ellipse plots show the KEGG pathway ID, the rhombus plots the target gene, and the green plots down-regulated genes. **(D)** PPI network analysis of the OMRGs. The nodes represent the OMRGs in the PPI network, and the lines show the interaction among the OMRGs. The size and color of nodes are associated with the significance degree of proteins, while the thickness of lines is proportional to the strength of interactions among proteins.

The biological function of the DEGs can be predicted by GO functional annotation analysis which depicts three biological functions including biological process, cellular component, and molecular function (Ashburner et al., 2000). GO analysis was performed using the online tool DAVID. The results showed biological process of the OMRGs enriched in positive regulation of transcription from RNA polymerase II promoter, bone mineralization, and regulation of immune response. The genes enriched at bone mineralization were S1PR1, TWIST1, and PHOSPHO1. For cellular component, OMRGs were assembled at membrane structure, such as bicellular tight junction, apical plasma membrane, and basolateral plasma membrane (Figure 2A). With regard to molecular function analysis, OMRGs were enriched in specific DNA binding, actin filament binding, and Erbb-3 class receptor binding (Figure 2A).

We performed DAVID to analyze the KEGG pathways of the OMRGs. Results showed that OMRGs were enriched in natural killer cell mediated cytotoxicity and antigen processing and presentation, especially pathways associated with the antigen processing and presentation (Figures 2B,C).

Protein-protein interaction networks were constructed using DEGs and protein interaction information obtained from STRING database. Results showed that there were 30 nodes and 30 edges in PPI network of OMRGs. Among this PPI network of OMRGs, erbb2 receptor tyrosine kinase 2 (ERBB2), twist family basic helix-loop-helix (bHLH) transcription factor 1 (TWIST1), Nanog homeobox (NANOG) were the top 3 core proteins according to the degree of distribution, closeness centrality, and betweenness centrality (Figure 2D and Supplementary Table S2).

### TWIST1 Up-Regulation Might Be Associated With Macrophages Accumulation at Infection Site

**FIGURE 3:**
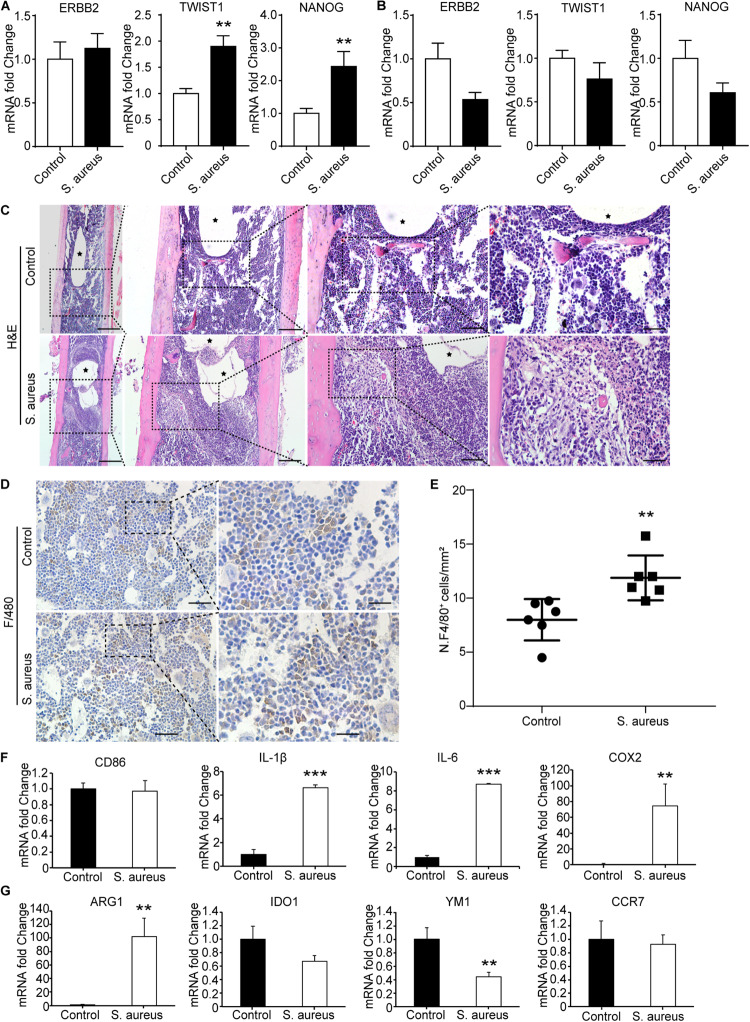
Macrophages accumulate around the infected implant in bone marrow of osteomyelitis mice. **(A,B)** qPCR analysis of the mRNA expression of TWSIT1, NONAG, and ERBB2 in bone marrow cells **(A)** and in peripheral blood cells **(B)** from *S. aureus* osteomyelitis mice and control group. *n* = 6/group. **(C)** H&E staining for the bone from mice with *S. aureus* infected implant and control mice. Scale bars form left to right represent 500, 200, 100, and 20 μm, respectively. Black stars represent implant track. *n* = 6/group. **(D)** Representative images of immunohistochemistry staining for F4/80 in the bone of *S. aureus* infected mice and control mice. **(E)** Quantitative analysis of the number of F4/80^+^ cells around an implant (*N*. F4/80^+^/mm^2^). Scale bars form left to right represent 100 and 20 μm, respectively. *N* = 5/group, **p < 0.01. **(F,G)** qPCR analysis of the mRNA expression of marker genes associated with macrophage polarization in bone marrow cells. *n* = 6/group. *p < 0.05, **p < 0.01, ***p < 0.0001, and Mann–Whitney *U*-test.

As TWIST1 was the most up-regulated gene in OMRGs of GPL96 platform, NANOG and ERBB2 were closely associated with TWIST1, and they were top 3 core proteins as well in PPI network, we established *S. aureus* osteomyelitis model, and evaluated the mRNA expression of these three genes in peripheral blood cells and bone of *S. aureus*-infected mice. Results showed that TWIST1 and NANOG were significantly up-regulated in bone marrow cells (Figure 3A), but not in peripheral blood cells (Figure 3B), of *S. aureus* osteomyelitis mice.

TWIST1 was reported to be involved in tumor cells migration (Xu et al., 2017; Gou et al., 2018). As macrophages and neutrophils are the main phagocytes to defend *S. aureus* infection in early infection stage (Miller and Cho, 2011), we speculate that *S. aureus* infection may stimulate cells migration by up-regulating the expression of TWIST1. As shown in H&E staining results, there were a lot of macrophages accumulated around the infected implant on day 3 after infection (Figure 3C). Consistent with the histochemistry results, immunohistochemistry staining for F4/80, a marker for macrophage, also showed significantly increased positive staining (Figures 3D,E).

To further evaluate the changes of macrophage at infection site in bone marrow, the mRNA expression levels of CD86, IL-1β, IL-6, and COX2, marker genes of the classically activated macrophages (M1), and ARG1, IDO1, YM1, and CCR7, marker genes of the alternatively activated macrophages (M2; Parisi et al., 2018), were evaluated in the bone marrow from *S. aureus* osteomyelitis mice. Results showed that the mRNA expression levels of IL-1β, IL-6, and COX2 were dramatically up-regulated, while YM1 expression levels were down-regulated (Figures 3F,G). All these data indicate that the up-regulated expression of TWIST1 might be associated with macrophages accumulation at infection site.

### TWIST1 Is Activated in Macrophages Around the Infection Site

**FIGURE 4:**
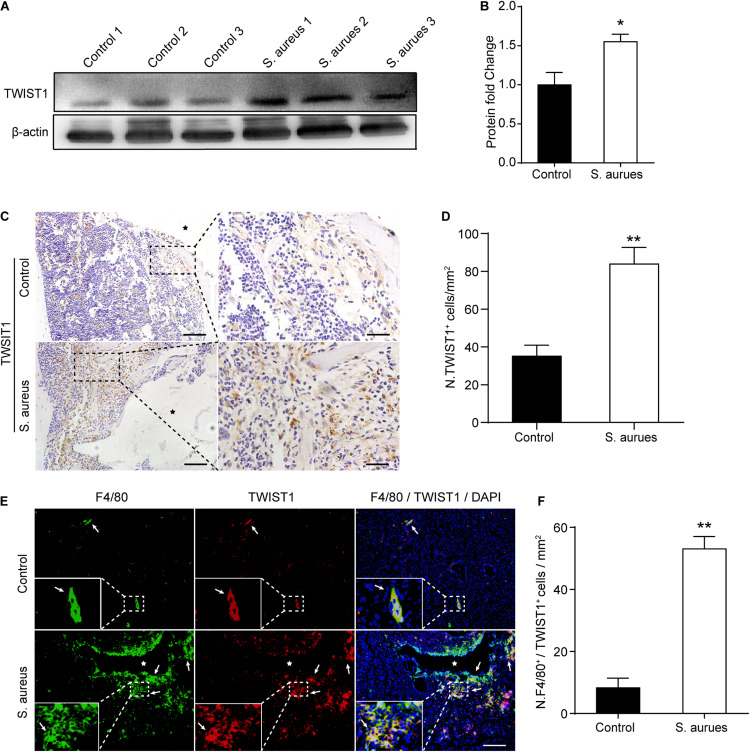
*S. aureus* infection activates the expression of TWIST1 in macrophages. **(A,B)** Western blotting analysis **(A)** and quantification **(B)** of TWIST1 expression in the bone marrow from *S. aureus* infected bone and control bone. *n* = 6/group. **(C)** Representative images of immunohistochemistry staining for TWIST1 in bone from *S. aureus* infected mice and control mice. Scale bars form left to right represents 100 μm and 20 μm, respectively, and stars represent the implant track. **(D)** Quantitative analysis of the number of TWIST1^+^ cells per tissue area around implant (*N*. TWIST1^+^/mm^2^). *n* = 6/group. **(E)** Representative images of double-immunofluorescence staining for TWIST1 and F4/80 in bone from *S. aureus* infected mice and control mice. Scale bar represents 100 μm. Stars locate the implant track white arrows point to positive stained cells. **(F)** Quantitative analysis of the number of F4/80^+^TWIST1^+^ cells per tissue area around implant (N. F4/80^+^TWIST1^+^/mm^2^). *n* = 4/group. *p < 0.05, **p < 0.01, and Mann–Whitney *U*-test.

To confirm the activated expression of TWIST1 in *S. aureus* osteomyelitis, protein was harvested from mice bone marrow at day 3 after infection. Consistent with the up-regulated expression level in mRNA, the protein level of TWIST1 was significantly increased in bone marrow infected by *S. aureus* (Figures 4A,B). To further verify this finding, we performed immunohistochemistry staining. Results showed a much higher amount of TWIST1-positive staining around the infection site than that in the control group (Figures 4C,D).

As macrophages were recruited around the infection site and TWIST1 was highly expressed around the infection, we speculated that the expression of TWIST1 might be up-regulated in macrophages around infection. Double-immunofluorescence of F4/80 and TWIST1 results demonstrated dramatically increased intensity of F4/80^+^TWIST1^+^ staining (Figures 4E,F).

### TWIST1 May Be Associated With Macrophage Polarization in Response to *S. aureus* Infection

**FIGURE 5:**
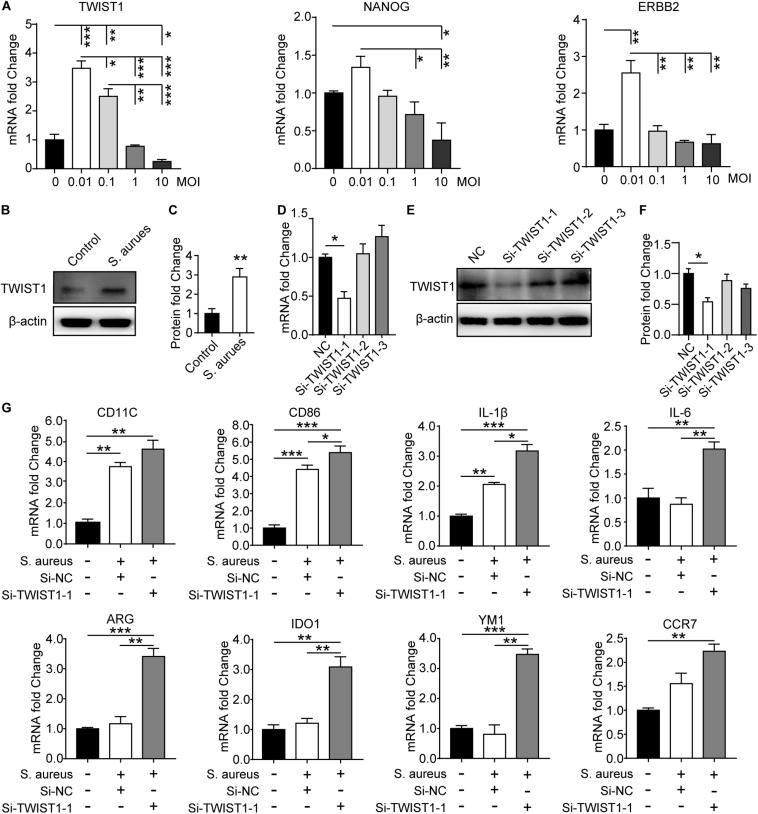
*S. aureus* infection activates the expression of TWIST1 in Raw 264.7 cells. **(A)** qPCR analysis of the mRNA expression of TWIAT1, NANOG, and ERBB2 in Raw 264.7 cells in response to *S. aureus* infection with MOI [the ratio of the *S. aureus* colonies number (CFU) to the Raw 264.7 cells number] at 0.01, 0.1, 1.0, and 10. ANOVA followed by Dunnett’s test, *n* = 3/group. **(B)** Immunoblot and **(C)** quantitative analysis of TWIST1 in Raw 264.7 cells after infection. Two-tailed Student’s *t* test, *n* = 3/group. **(D)** qPCR analysis of the effect of siRNA fragments on the mRNA expression of TWIST1 in Raw 264.7 cells. ANOVA followed by Dunnett’s test, *n* = 3/group. **(E)** Immunoblot and **(F)** quantitative analysis of the effect of siRNA fragments on the protein expression of TWIST1 in Raw 264.7 cells. ANOVA followed by Dunnett’s test, *n* = 3/group. **(G)** qPCR analysis of the effect of knocking down TWIST1 on the mRNA expression of marker genes for macrophage polarization in Raw 264.7 cells after *S. aureus* infection. ANOVA followed by Dunnett’s test, *n* = 3/group. *p < 0.05, **p < 0.01, and ***p < 0.001.

To verify the effect of *S. aureus* infection on the expression of TWIST1 in macrophages, Raw 264.7 cells were infected with *S. aureus* at different MOIs [the concentration of *S. aureus* (CFU) to Raw 264.7 cells, MOI = 10, 1, 0.1, 0.01]. In accordance with the result *in vivo*, we found significantly up-regulated expression of TWIST1. The mRNA level and protein level of TWIST1 decreased gradually with the severity of infection (Figure 5A and Supplementary Figures S2A,B). We believed that this phenomenon might be associated with increased cell mortality due to high MOI of infection (Supplementary Figures S2C,D). Additionally, western blotting results confirmed the up-regulated expression of TWIST1 in macrophages after infection with *S. aureus* at 0.01 MOI (Figures 5B,C). To explore the role of TWIST1 in Raw 264.7 cells after infection, transient RNA interference (Si-TWIST1) was performed to screen out the best interference effect in mRNA level (Figure 5D) and protein level (Figures 5E,F). The marker genes for M1 macrophage were dramatically up-regulated in Raw 264.7 macrophage cells *S. aureus* infection (Figure 5G). Further, the mRNA expression levels of marker genes associated with macrophage polarization were considerably changed after knocking down TWIST1. Results showed that the mRNA expression levels of ARG, IDO1 and YM1, marker genes for M2 macrophages, were dramatically up-regulated (Figure 5G). The data indicate that TWIST1 might be associated with macrophage polarization in response to *S. aureus* infection.

### TWIST1 May Regulate the Migration and Phagocytosis of Macrophage

**FIGURE 6:**
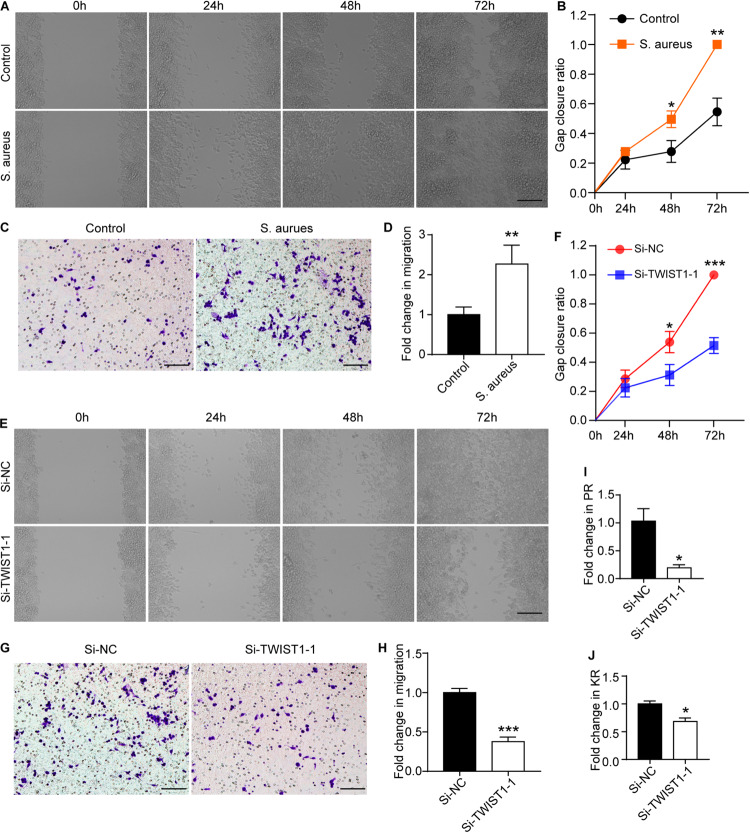
TWIST1 mediates the migration and phagocytosis of macrophage in response to *S. aureus* infection. Wound-healing assay was used to evaluate the effect of *S. aureus* on macrophage migration. After 1 × 10^6^ cells were seeded in 6-well plate and cultured for 12 h, they were infected with *S. aureus* at 0.01 MOI. **(A)** Representative images for wound-healing assay and **(B)** quantitative analysis of gap closure ratio of Raw 264.7 cells at 0, 24, 48, and 72 h after *S. aureus* infection. Scale bar represents 100 μm. **(C)** Transwell-based migration assays and **(D)** quantitative analysis of the migrated cells of Raw 264.7 cells at 24 h after *S. aureus* infection at 0.01 MOI. Scale bar represents 100 μm. **(E)** Wound-healing assay and **(F)** quantitative analysis of gap closure ratio of Raw 264.7 cells at 0, 24, 48, and 72 h after *S. aureus* infection. Scale bar represents 100 μm. NC represents negative control. Cells were transfected with negative random fragments. **(G)** Transwell-based migration assays and **(H)** quantitative analysis of the migrated cells of Raw 264.7 cells at 24 h after *S. aureus* infection. Scale bar represents 100 μm. Quantitative analysis of the effect of knocking down TWIST1 on Phagocytosis rate (PR; **I**) and killing rate (KR; **J**) of Raw 264.7 cells in response to *S. aureus* infection. *p < 0.05, **p < 0.01, ***p < 0.001, and Two-tailed Student’s *t* test, *n* = 3/group.

As there were more macrophages at the infection site, we speculated that TWIST1 might be associated with the migration and recruitment of macrophages to the infection sites in marrow. To prove this speculation, the wound-healing assay and transwell-based migration assays were performed. Interestingly, the Raw 264.7 cells infected by *S. aureus* migrated faster than control cells treated with vehicle (Figures 6A–D), and the down-regulation of TWIST1 inhibited the migration of Raw 264.7 cells in response to *S. aureus* (Figures 6E,H). In addition, knocking-down TWIST1 significantly reduced the phagocytosis rate and killing rate of Raw 264.7 in response to *S. aureus* infection (Figures 6I,J).

### *S. aureus* Promotes Macrophage Migration Through P65/TWIST1/MMP9/MMP13 Axis

**FIGURE 7:**
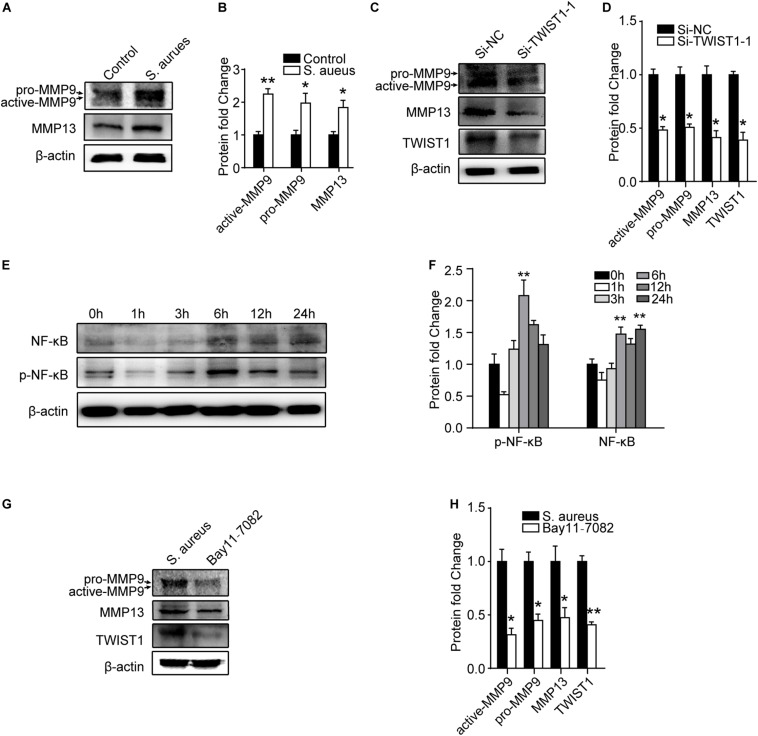
NF-κB/TWIST1 mediates the effect of *S. aureus* on macrophages. **(A)** Immunoblot and **(B)** quantitative analysis of MMP9 and MMP13 expression in Raw 264.7 cells in response to *S. aureus* infection. Two-tailed Student’s *t* test, *n* = 3/group. **(C)** Immunoblot and **(D)** quantitative analysis of the effect of knocking down TWIST1 on the expression of MMP9 and MMP13 in Raw 264.7 cells after *S. aureus* infection. Two-tailed Student’s *t* test, *n* = 3/group. **(E)** Immunoblot and quantitative analysis **(F)** of NF-κB in Raw 264.7 cells at 0, 1, 3, 6, 12, and 24 h after *S. aureus* infection. ANOVA followed by Dunnett’s test, *n* = 3/group. **(G)** Immunoblot and quantitative analysis **(H)** of the effect of NF-κB inhibitor (Bay11-7082) on the expression of MMP9 and MMP13 in Raw 264.7 cells after *S. aureus* infection. Two-tailed Student’s *t* test, *n* = 3/group. *p < 0.05, and **p < 0.01.

It was reported that TWIST1 promoted extracellular matrix degradation and cells migration through upregulating matrix metallopeptidases especially MMP9 and MMP13 (Ding et al., 2019). We speculated that *S. aureus* infection might stimulate TWIST1 expression, thereby up-regulating MMP9 and MMP13 expression. Interestingly, we found that MMP9 (active-MMP9 and pro-MMP9), and MMP13 in Raw 264.7 cells were significantly up-regulated in response to *S. aureus* infection (Figures 7A,B). Furthermore, knocking-down the TWIST1 dramatically suppressed the expression of MMP9 and MMP13 (Figures 7C,D).

As NF-κB (P65) was reported to be associated with the expression of TWIST1 (Li C. W. et al., 2012; Li S. et al., 2012; Liu et al., 2015), we evaluated the expression of NF-kB after *S. aureus* infection. Activation of NF-kB was observed in a time-dependent manner 6 h after *S. aureus* infection (Figures 7E,F). To further evaluate the role of NF-kB in *S. aureus* induced expression of TWIST1/MMP9/13, P65 signaling inhibitor, Bay 11–7082 (10 μM, pretreatment 6 h) was used to block NF-κB when infected by *S. aureus*. Results showed that Bay11-7082 significantly suppressed the protein levels of TWIST1, MMP9, and MMP13 after *S. aureus* infection (Figures 7G,H).

## Discussion

*Staphylococcus aureus* is a leading cause for bloodstream infection-induced osteomyelitis in children (Street et al., 2015). Understanding the pathogenesis of early host response is important in prevention and elimination of *S. aureus* osteomyelitis. In the present study, bioinformatics was used to investigate the system-level responses of PBMCs to *S. aureus* osteomyelitis. ERBB2, TWIST1, and NANOG were screened out as the most important OMRGs. We found dramatically up-regulated expression of TWIST1 and macrophage recruitment at the infection site of implant-related osteomyelitis mice model. Further, we demonstrated that *S. aureus* stimulated polarization, migration and phagocytosis of macrophage by up-regulating NF-κB/TWIST1 signaling.

Gene ontology analysis revealed that biological process of the OMRGs was enriched in the genes enriched at bone mineralization, like down-regulated S1PR1, and up-regulated TWIST1 and PHOSPHO1. Intriguingly, these genes have been shown to target bone formation and bone matrix mineralization (Isenmann et al., 2009; Dillon et al., 2019; Lewis et al., 2019; Xiao et al., 2019). It is likely that there are multiple genes involved in infection-induced changes in bone remodeling, but the relative importance of these distinct genes in bone remodeling during infection remains to be determined.

Protein-protein interaction network analysis of OMRGs revealed changes of 3 core proteins, down-regulated ERBB2, up-regulated TWIST1, and NANOG. TWIST1 is a bHLH transcription factor, over-expression of which stimulates proliferation of bone marrow mononuclear cells, aggravates the inflammatory balance (Wang et al., 2015; Mahadik et al., 2018), and is related to cells migration (Xu et al., 2017; Gou et al., 2018; Zhou et al., 2019). NANOG is a key transcription factor for pluripotency of CD14^+^ monocytes, involved in immune response, repair and regeneration of damaged tissue (Seta and Kuwana, 2010). ERBB2, the second ErbB-family member, is ubiquitously expressed and commonly altered in metastatic breast, prostate and ovarian cancers (Arteaga and Engelman, 2014). A recent study reveals a critical function of ERBB2 in innate immune modulation. ERBB2 inhibitor or ablation of ERBB2 expression leads to a dramatic suppression of HSV-1 infection (Wu et al., 2019). Therefore, up-regulation of TWIST1 and NANOG and down-regulation of ERBB2 might indicate activation of immune cells in response to *S. aureus* osteomyelitis.

It is somewhat unexpected that the up-regulated expression of TWIST1 and NANOG was only found in the cells from bone marrow but not in those from blood and the expression of ERBB2 was only down-regulated in the cells from blood in implant-associated osteomyelitis mice models. This might have been because the implant-associated infection model we used to observe the genes expression was only at an early stage of *S. aureus* infection when the infection had not spread to elsewhere. It will be interesting to determine in a future study the changes in expression of these genes at different infection stages in mice models of hematogenous and implant-associated osteomyelitis. In addition, the healthy controls and osteomyelitis patients were at a median age of 6–7.5 years old in the PBMCs samples for the transcriptional data whereas the implant-associated infection mice were 8 weeks old in the OMRGs verification, which is equivalent to about 20 years of age in human (Dutta and Sengupta, 2016). Therefore, age difference might have been another major cause leading to the above discrepancy.

Macrophages are actively involved in clearance of bacteria, thereby providing the first line of defense against invading pathogens (Motwani and Gilroy, 2015). Our data provided evidence that the amount of macrophage was significantly increased around *S. aureus*-infected implant. Additionally, we found up-regulated expression of TWIST1 in macrophage around *S. aureus*-infected implant. TWIST1 has been found to be involved in promoting cell migration through up-regulating the expression of MMPs in cancer cells (Xu et al., 2017; Gou et al., 2018; Zhou et al., 2019). However, little is known about the role of TWIST1 in regulating macrophage function under *S. aureus* infection. Our data reveal that *S. aureus* infection may stimulate the expression of TWIST1 which up-regulates the expression of MMP9 and MMP 13, thereby promoting the migration of macrophages toward the infection sites for scavenging *S. aureus*.

It is known that the balance between classically activated (M1) macrophages and alternatively activated (M2) macrophages governs the fate of an organ in infection. M1 macrophages release cytokines which can cause tissue damage while M2 macrophages secret factors to suppress the inflammation and promote tissue remodeling (Shapouri-Moghaddam et al., 2018). NF-κB is considered to be a member of pro-inflammatory family of main transcription factors in inflammatory processes. It participates in M1 macrophage polarization induced by various stimulations (Gao et al., 2018; Genard et al., 2018). Consistent with their reports, our data demonstrate that NF-κB pathway activation is associated with TWIST1 expression and M1 polarization in response to *S. aureus* infection. Our data suggest that *S. aureus* may activate TWIST1 expression through NF-κB, thereby promoting macrophage migration and M1 polarization. Of note, TWIST1 may limit inflammation through a negative feedback loop that represses NF-κB activity (Šošić et al., 2003; Merindol et al., 2014). It is possible that TWIST1 and NF-κB may form a negative regulatory loop in modulating macrophage reaction during *S. aureus* infection.

This study has 2 limitations. First, we did not further investigate the role of up-regulated NANOG and down-regulated ERBB2 in *S. aureus* infection. Additional further studies are warranted to determine in which cells NANOG and ERBB2 are regulated and their roles in *S. aureus* infection. Secondly, we only evaluated the mRNA expression of 3 core genes obtained from PPI network analysis of OMRGs. It will be important in the future studies to verify more OMRGs and their roles in animal osteomyelitis models.

In conclusion, we identified the DEGs of osteomyelitis caused by *S. aureus* infection based on publicly available transcriptional dataset GSE16129 using bioinformatic analysis. Our *in vivo* and *in vitro* data demonstrate that NF-κB/TWIST1 is necessary for *S. aureus* infection-induced macrophage migration and phagocytosis. Our study highlights a previously unknown fundamental function of TWIST1 in the early stages of the innate immune response to *S. aureus* infection in bone.

## Data Availability Statement

The datasets generated for this study can be found in the Gene Expression Omnibus (GEO; https://www.ncbi.nlm.nih.gov/gds/?term=gse16129), GSE16129 (Ardura et al., 2009).

## Ethics Statement

The publicly available GSE16129 dataset involving human participants were reviewed and approved by Institutional Review Boards of the University of Texas Southwestern Medical Center and Children’s Medical Center of Dallas (IRB #0802-447) and Baylor Institute of Immunology Research (BIIR, IRB #002-141). Written informed consent to participate in this study was provided by the participants’ legal guardian/next of kin (Ardura et al., 2009). The animal study was reviewed and approved by Animal experiment ethics committee of Nanfang hospital.

## Author Contributions

XZ and BY conceived and designed the study. YW performed the bioinformatics analyses and drafted the manuscript and carried out the experiment. YL and CC established a model of osteomyelitis. YW and PZ performed *in vitro* experiments. PC and HW drafted sections of the manuscript. PZ, KL, YD, and JQ composed the figures in this manuscript. XZ and BY revised and approved the manuscript.

## Conflict of Interest

The authors declare that the research was conducted in the absence of any commercial or financial relationships that could be construed as a potential conflict of interest.

## Footnotes

1 http://www.ncbi.nlm.nih.gov/geo/

2 https://www.r-project.org/

3 www.bioconductor.org

4 http://bioinfogp.cnb.csic.es/tools/venny/index.html

5 http://david.abcc.ncifcrf.gov

6 http://www.string-db.org/

## Supplementary Material

FIGURE S1 Sample data preprocessing. **(A,B)** Box plots of the expression value before and after normalization of GPL 96 and GPL97. Blue plots represent the unnormalized expression value, the red pots the normalized expression value, the horizontal axis the samples, and the vertical axis the expression value. **(C,D)** Volcano plots for DEGs of platform GPL96 and GPL97. Plots of the relationship between -log10 (adjusting *p* value) and log2 (fold change) for Ctrl vs OFI, Ctrl vs OMI, and Ctrl vs SI. Red plots represent the up-regulated or down-regulated genes that log2 (fold change) >1 or <-1, also -log10 (adjusted *P*-value) >1.301.

FIGURE S2 To determine the optimal MOI for *S. aureus* infection of Raw264.7 macrophages **(A)** Immunoblot and quantitative analysis **(B)** of TWIST1 in Raw 264.7 cells after *S. aureus* infection in different MOIs. **(C)** Trypan blue staining of Raw 264.7 cells after *S. aureus* infection for 24 h in different MOIs. **(D)** Quantitative analysis of viability (%Ctrl) of Raw 264.7 cells after *S. aureus* infection for 24 h in different MOIs. ANOVA followed by Dunnett’s test, *n* = 3/group. ^∗^*p* < 0.05, ^∗∗^*p* < 0.01, and ^∗∗∗^*p* < 0.001.
