# Corrigendum: NF-κB/TWIST1 Mediates Migration and Phagocytosis of Macrophages in the Mice Model of Implant-Associated *Staphylococcus aureus* Osteomyelitis

**DOI:** 10.3389/fmicb.2020.01775

**Published:** 2020-08-13

**Authors:** Yutian Wang, Yihuang Lin, Caiyu Cheng, Pengyu Chen, Ping Zhang, Hangtian Wu, Kaiqun Li, Ye Deng, Jikun Qian, Xianrong Zhang, Bin Yu

**Affiliations:** ^1^Department of Orthopaedics, Nanfang Hospital, Southern Medical University, Guangzhou, China; ^2^Guangdong Provincial Key Laboratory of Bone and Cartilage Regenerative Medicine, Nanfang Hospital, Southern Medical University, Guangzhou, China

**Keywords:** *Staphylococcus aureus*, osteomyelitis, macrophage, bioinformatics, bone remodeling

In the original article, there was an error. According to the “Macrophage phagocytosis” part in the Materials and Methods of the manuscripts, N0 stood for the number of colonies formed by phagocytized bacteria by macrophage after 1 h infection, and N1 stood for the number of colonies formed by survival bacteria inside macrophage 1 h after elimination of extracellular bacteria. The rate of phagocytosis was calculated as N0/(2 × 10^5^) (%), and the rate of bacterial killing was calculated as (N0-N1)/N0 (%), not (N1-N0)/N0 (%).

A correction has been made to ***Materials and Methods***, sub-section ***Macrophage phagocytosis***. The corrected paragraph appears below.

2 × 10^5^ Raw 264.7 cells were infected with *S. aureus* at a MOI of 0.01 for 1 h, following by treatment with 20 μg/ml gentamicin for 30 min to kill extracellular bacteria. Cells were washed with PBS for three times, followed by lysis with 0.2% Triton. The cell lysis mixture was cultured on TSB agar plates overnight at 37°C. Bacteria colonies were counted and set as N0. To evaluate the phagocytosis of macrophage, after extracelluar bacteria eliminated, cells were allowed to grow in fresh 10% FBS medium for an additional 1 h. Then cells were lysed and cell lysis mixture was grown on TSB agar plates, and bacteria colonies were counted and set as N1. The rate of phagocytosis was calculated as N0/(2 × 10^5^) (%), and the rate of bacterial killing was calculated as (N0-N1)/N0 (%).

The authors apologize for this error and state that this does not change the scientific conclusions of the article in any way. The original article has been updated.

