# Supplementary figures and images for: NF-κB/TWIST1 Mediates Migration and Phagocytosis of Macrophages in the Mice Model of Implant-Associated Staphylococcus aureus Osteomyelitis
Source: Front Microbiol. 2020 Jun 12;11:1301. doi: 10.3389/fmicb.2020.01301 (PMC7304240; doi:10.3389/fmicb.2020.01301)

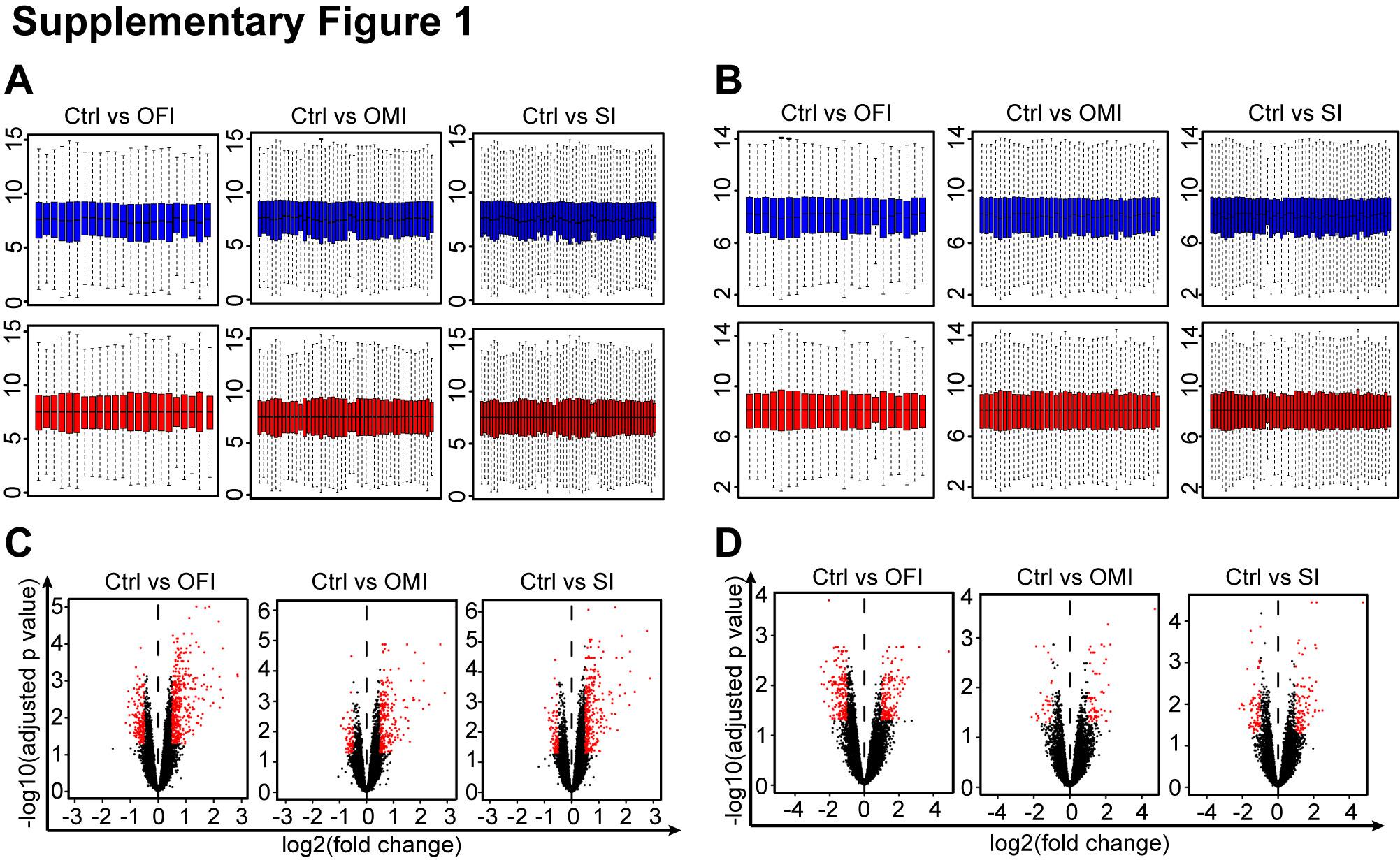

Supplement: FIGURE S1 — Sample data preprocessing. (A,B) Box plots of the expression value before and after normalization of GPL 96 and GPL97. Blue plots represent the unnormalized expression value, the red pots the normalized expression value, the horizontal axis the samples, and the vertical axis the expression value. (C,D) Volcano plots for DEGs of platform GPL96 and GPL97. Plots of the relationship between -log10 (adjusting p value) and log2 (fold change) for Ctrl vs OFI, Ctrl vs OMI, and Ctrl vs SI. Red plots represent the up-regulated or down-regulated genes that log2 (fold change) >1 or <-1, also -log10 (adjusted P-value) >1.301. [file Image_1.TIF]

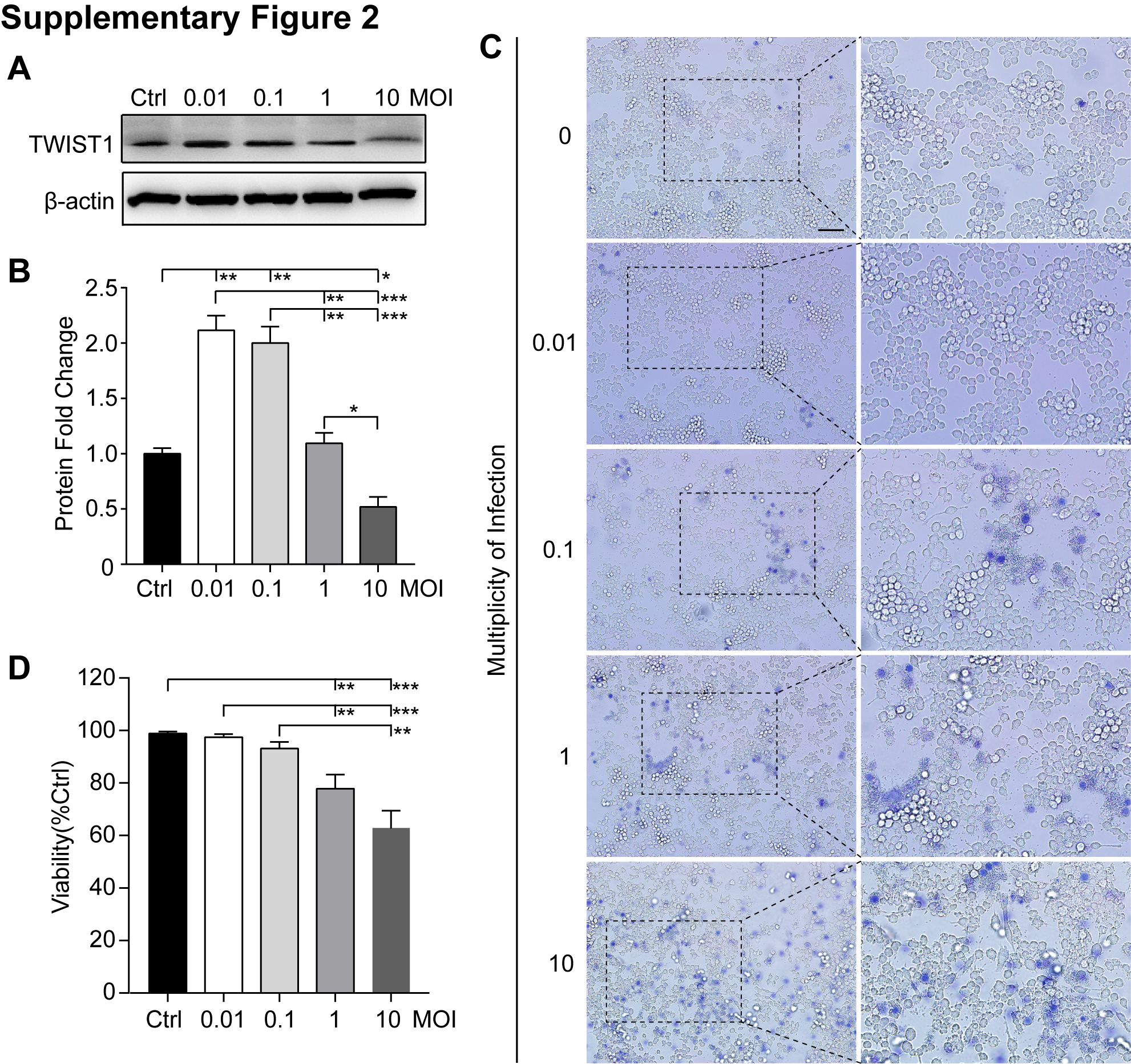

Supplement: FIGURE S2 — To determine the optimal MOI for S. aureus infection of Raw264.7 macrophages (A) Immunoblot and quantitative analysis (B) of TWIST1 in Raw 264.7 cells after S. aureus infection in different MOIs. (C) Trypan blue staining of Raw 264.7 cells after S. aureus infection for 24 h in different MOIs. (D) Quantitative analysis of viability (%Ctrl) of Raw 264.7 cells after S. aureus infection for 24 h in different MOIs. ANOVA followed by Dunnett’s test, n = 3/group. ∗p < 0.05, ∗∗p < 0.01, and ∗∗∗p < 0.001. [file Image_2.TIF]
