# Supplementary material for: NF-κB/TWIST1 Mediates Migration and Phagocytosis of Macrophages in the Mice Model of Implant-Associated Staphylococcus aureus Osteomyelitis
Source: Front Microbiol. 2020 Jun 12;11:1301. doi: 10.3389/fmicb.2020.01301 (PMC7304240; doi:10.3389/fmicb.2020.01301)
Supplement: Supplementary file 3 [file Table_1.DOCX]

Supplement Table 1. Primers used for quantitative real‐time polymerase chain reaction.

| Genes | Forward primers | Reverse primers | Accession no. |
| --- | --- | --- | --- |
| TWIST1 | 5′‐GGACAAGCTGAGCAAGATTCA-3' | 5'-CGGAGAAGGCGTAGCTGAG-3' | NM_011658.2 |
| NANOG | 5'-TCTTCCTGGTCCCCACAGTTT-3' | 5'-GCAAGAATAGTTCTCGGGATGAA-3' | NM_001289828.1 |
| ERBB2 | 5'-GAGACAGAGCTAAGGAAGCTGA-3' | 5'-ACGGGGATTTTCACGTTCTCC-3' | NM_001003817.1 |
| CD86 | 5'-TGTTTCCGTGGAGACGCAAG-3' | 5'-TTGAGCCTTTGTAAATGGGCA-3' | NM_019388.3 |
| IL-1β | 5'-GCAACTGTTCCTGAACTCAACT-3' | 5'-ATCTTTTGGGGTCCGTCAACT-3' | NM_008361.4 |
| IL-6 | 5'-TAGTCCTTCCTACCCCAATTTCC-3' | 5'-TTGGTCCTTAGCCACTCCTTC-3' | NM_012589.2 |
| COX2 | 5’-TCAGGCAGTATAATCCAAAGATGGT-3’ | 5’-AGTCTGGCTTATATCCAACACTTCG-3’ | YP_001686701.1 |
| ARG1 | 5'-CTCCAAGCCAAAGTCCTTAGAG-3' | 5'-AGGAGCTGTCATTAGGGACATC-3' | NM_007482.3 |
| IDO1 | 5‘-CAATCAAAGCAATCCCCACTG-3’ | 5‘-AAAACGTGTCTGGGTCCAC-3’ | NM_001293690.1 |
| YM1 | 5‘-GTACAAGATCCCTGAACTGTCTC-3’ | 5‘-TCCACATTGAGATCAGCACTC-3’ | NM_009892.3 |
| CCR7 | 5'-TGTACGAGTCGGTGTGCTTC-3' | 5'-GGTAGGTATCCGTCATGGTCTTG-3' | NM_001301713.1 |
| CD11C | 5'-CTGGATAGCCTTTCTTCTGCTG-3' | 5'-GCACACTGTGTCCGAACTCA-3' | NM_001363984.1 |
