# Supplementary material for: NF-κB/TWIST1 Mediates Migration and Phagocytosis of Macrophages in the Mice Model of Implant-Associated Staphylococcus aureus Osteomyelitis
Source: Front Microbiol. 2020 Jun 12;11:1301. doi: 10.3389/fmicb.2020.01301 (PMC7304240; doi:10.3389/fmicb.2020.01301)
Supplement: Supplementary file 4 [file Table_2.DOCX]

Supplement Table 2. The top 15 core proteins were identiﬁed in topological analysis of the PPI network of the OMRGs.

| DEGs | Degree | Betweenness Centrality | Closeness Centrality | Gene name | Expressing change |
| --- | --- | --- | --- | --- | --- |
| OMRGs | 7 | 0.74145299 | 0.61904762 | ERBB2 | Down |
|  | 4 | 0.12393162 | 0.46428571 | TWIST1 | Up |
|  | 3 | 0 | 0.43333333 | NANOG | Up |
|  | 3 | 0 | 0.43333333 | ALDH1A1 | Down |
|  | 3 | 0.28205128 | 0.37142857 | NCR3 | Down |
|  | 3 | 0.03205128 | 0.35135135 | EGR1 | Up |
|  | 3 | 0.66666667 | 1 | PLD1 | Up |
|  | 2 | 0.15384615 | 0.41935484 | ACPP | Up |
|  | 2 | 0.66666667 | 0.75 | IL7 | Down |
|  | 2 | 0.0534188 | 0.43333333 | ETS1 | Down |
|  | 2 | 0.38461538 | 0.48148148 | LIN7A | Up |
|  | 2 | 0 | 0.2826087 | KIR2DL1 | Down |
|  | 2 | 0 | 0.2826087 | KIR3DL1 | Down |
|  | 2 | 0.66666667 | 0.75 | S1PR1 | Down |
|  | 2 | 0.02136752 | 0.36111111 | MTA2 | Up |
